# The reasoning criminal vs. Homer Simpson: conceptual challenges for crime science

**DOI:** 10.3389/fnhum.2013.00682

**Published:** 2013-10-23

**Authors:** Noémie Bouhana

**Affiliations:** Department of Security and Crime Science, University College LondonLondon, UK

**Keywords:** crime science, situational crime prevention, rational choice, decision-making, theory

## Abstract

A recent disciplinary offshoot of criminology, crime science (CS) defines itself as “the application of science to the control of crime.” One of its stated ambitions is to act as a cross-disciplinary linchpin in the domain of crime reduction. Despite many practical successes, notably in the area of situational crime prevention (SCP), CS has yet to achieve a commensurate level of academic visibility. The case is made that the growth of CS is stifled by its reliance on a model of decision-making, the Rational Choice Perspective (RCP), which is inimical to the integration of knowledge and insights from the behavioral, cognitive and neurosciences (CBNs). Examples of salient developments in the CBNs are provided, as regards notably multiple-system perspectives of decision-making and approaches to person-environment interaction. Short and long-term benefits of integration for CS are briefly outlined.

## A very short introduction to crime science

A recent disciplinary offshoot of criminology, crime science (CS) defines itself as “the application of science to the control of crime” (Laycock et al., 2005; Laycock, 2008:149). Problem-driven, CS is chiefly concerned with the design of social and technological systems in service to the needs of stakeholders and end-users—be they industry, government, security agencies, or the general public. Underpinning CS and its preferred approach to crime reduction, situational crime prevention (SCP), is the premise that crime is best tackled by targeting its immediate causes. This focus on proximate factors is intentionally lopsided. While the necessary conditions of crime are defined as the intersection in time and space of a motivated offender and a suitable target in the absence of a capable guardian, relatively little attention has been paid to the “offender” part of the equation. CS digs its philosophical roots in the 18th Century Classical School, whereby Man is understood as an essentially self-interested animal driven by desires which he seeks to fulfill while incurring the least amount of effort. Susceptibility to temptation is thus taken as a given and CS looks to situational control—the removal of temptations—as the most promising crime reduction strategy. “Opportunity makes the thief”: remove the opportunity, increase the effort and reduce the rewards of offending, and the crime will be prevented (Clarke, 2012).

The effectiveness of this approach has been demonstrated against a diverse range of crime problems. The promise of technological solutions and an emphasis on practical problem-solving have been popular with law enforcement agencies, and the claim was made that CS would soon eclipse criminology departments within universities (Clarke, 2004). However, CS has yet to achieve commensurate visibility in the academic sphere. This paper contends that the conceptual limitations of CS’s standard model of decision-making, the Rational Choice Perspective (RCP), as well as the discipline’s largely “bottom-up” research programme, hold it back from fulfilling its stated ambition to act as a cross-disciplinary linchpin (Laycock et al., 2005). The case is made that CS must look to developments in the cognitive, behavioral and neurosciences (henceforth, Cognitive and Neurosciences (CBNs)) to address RCP’s shortcomings. Examples of developments which suggest potential for integration are provided. In conclusion, the benefits of integration are further outlined.

## The case for “bounded” parsimony

It is not possible to leave the offender out of crime prevention altogether. In order to “increase effort” and “reduce rewards”, a model of criminal decision-making is needed. For this purpose, the fathers of SCP adopted the RCP (Clarke et al., 1985; Cornish et al., 2008). As presented, RCP is not a theory *per se*, but a heuristic device, a “good enough” conceptual model which provides a schematic understanding of how offenders make decisions—evaluating, to the best of their abilities, the costs and benefits of their actions. Armed with this basic understanding, the crime controller can design an array of situational techniques to influence the offender’s decisional process away from crime (Smith and Clarke, 2012).

While RCP has met with notable success as an engineering heuristic, it has fallen short as a model of offender decision-making (Wortley et al., 2013). Although the framework acknowledges, on the one hand, the less-than-rational aspects of offender decision-making—criminal rationality is described as “bounded”—it implies, on the other, that the problem isn’t worth agonizing over: a parsimonious, *as-if* model, unencumbered by the vagaries of human affect and cognition, should serve the crime controllers well enough (Smith and Clarke, 2012). As Wortley et al. (2013) observes, this state of affairs has had the consequence of stifling theoretical development in CS, so much so that RCP has remained essentially static since the 1980s. One may take Wortley’s critique further and observe that other theoretical perspectives within the “family” of opportunity theories—notably, the Routine Activity Approach (Cohen and Felson, 1979)—have likewise remained relatively untouched. Opportunity theories are still, to a large extent, axiomatic statements rather than explanations of the causal processes which bring crime about (Wikström et al., 2011). This is illustrated by the oft-repeated claim that opportunities cause crime (Felson and Clarke, 1998); for it is not, of course, the opportunity which causes the crime, but its perception by the offender (the Thomas Theorem in action), among other processes: opportunities, whether provocations or temptations, are not criminal in themselves. To address this problem, some have proposed that the ecological concept of *affordance* (Gibson, 1979) should replace opportunity in CS parlance (Pease et al., 2006). However, affordance has yet to be integrated into the wider opportunity control framework. To take affordance on board, a model of criminal action is required which explains motivation in terms of the interaction between individual and situation, instead of postulating it as a given.

The move towards a more dynamic, interactionist model has been resisted, for fear that it would compromise RCP’s radical parsimony, a condition of its heuristic usefulness. Faced with evidence of the non-rational features of offender decision-making, the strategy has been to stretch the concept of “rationality” to encompass the new phenomena. Drives to criminal action are restated as factors in a cost-benefit analysis. Psychological rewards (e.g., excitement), moral emotions (e.g., guilt, shame), social inducements (e.g., status), psychobiological factors (e.g., addiction), and so on, are reinterpreted in “rational” terms (e.g., Clarke et al., 1997). This approach renders the model impregnable, but runs roughshod over Einstein’s admonition that theory should “make the irreducible basic elements as simple and as few as possible* without having to surrender the adequate representation of a single datum of experience”* (Einstein, 1934:165, emphasis added). The construct which explains everything explains nothing: the more phenomena is stuffed into the construct, the emptier it becomes. “Bounded” rather than radical parsimony would seem the more reasonable option.

## Drawbacks of “bottom-up” research

Calls to overhaul RCP and bring the offender back into SCP have been sounded in the past (Ekblom and Tilley, 2000; Wortley, 2001; Wortley et al., 2013), but have fallen on reluctant ears. New SCP techniques concerned with situational precipitators have been added to the catalogue (Cornish et al., 2003), falling far short of a conceptual shake-up. CS’s continuing identity struggle may explain this inertia: “science” moniker aside, CS is fundamentally an engineering discipline, with a self-confessed preference for short-term problem-solving (Laycock et al., 2005). At the outset, SCP was established as the technological framework most likely to deliver returns. A number of technological rules and design principles, most of them implicated in opportunity control, were identified, which produced reliable results. The discipline’s scientific programme was thus largely circumscribed to those research activities which provided a knowledge-base for the design of opportunity control technologies (broadly defined), or contributed to the testing, validation and refinement of those technological rules and design principles at the heart of the discipline.

Arguably, the crime scientist’s trademark question is, “So what?” (Laycock, 2012). If the topic is not self-evidently useful to crime control, it is not worth investigating. On the upside, this instrumental approach, whereby CS’s engineering ambitions dictate the discipline’s research activity, has produced reliable analytical tools and prevention technologies, which have achieved concrete gains in terms of crime reduction. On the downside, this relatively narrow research agenda has done little to encourage inquiry driven by “big questions”. Indeed, crime scientists have been known to take criminologists to task for studying the “wrong” kinds of causes and failing to be more problem-oriented (Clarke, 2004), as if only a finite number of scientific questions about crime were worth asking.

The concern is that this “bottom-up” research agenda has insularised CS from a wealth of knowledge in other disciplines, notably the CBNs, as much as it has impeded theoretical growth from within. Yet a field which looks to medicine as a desirable model of cross-disciplinarity (Laycock et al., 2005) needs a conceptual framework which *affords* (in Gibson’s sense of the word) disciplinary integration. Medicine and its parent disciplines share the foundations of a systemic (chemical, biological, psychosocial, ecological, and so on) understanding of the human organism and its environment. To achieve its stated goal, CS needs, if not a unified framework, then conceptual models which are not inimical to neighboring research programmes. As a first step, opportunity perspectives should clarify what they mean by “bounded rationality” and formulate explicit mechanisms of person-situation interaction (which will also necessitate a clear definition of “situation”; Snyder, 2013). Examples of developments in the CBNs may illustrate the value of integration.

## Enters Homer Simpson, stage right

The outsider looks on with envy at the effervescence which has characterized the growth and, increasingly, the integration of the CBNs in recent years. Given the breakneck speed of research in these domains, an overview isn’t attempted, but it is noteworthy that the surge of activity has often been accompanied, if not triggered, by an empirical challenge to single-factor (notably rationalist) models and theories.

In social psychology, dual-process models (Evans, 2003; Mischel et al., 2004; Kahneman et al., 2005; Kahneman, 2011) followed from observations that departures from classical rationality are an ubiquitous feature of human thinking (Kahneman et al., 1982; Kahneman, 2011). In moral psychology, dual models of moral judgment have likewise emerged which call into question the Kholbergian view of moral development, adopting instead an adaptationist perspective in which moral intuitions underpin moral judgment as much as moral reasoning, if not more so (Haidt, 2001; Greene and Gazzaniga, 2009; Cushman et al., 2010).

Of particular interest, given SCP’s original borrowing of the rational perspective from economics, has been the development of behavioral economics, which built upon social psychology’s insights to address commonly observed violations of the standard neo-classic model (Thaler, 1991; Mullainathan et al., 2001). As Camerer et al. (2004) put it, “At the core of behavioral economics is the conviction that increasing the realism of the psychological underpinnings of economic analysis will improve economics on its own terms—generating theoretical insights, making better predictions of field phenomena, and suggesting better policy.” The scientific gain, behavioral economists feel, is worth renouncing the seductive (i.e., simple and clear-cut), but ultimately misleading, solutions proposed by standard models. While neo-classical economics would like people to think like Mr. Spock, the average human being is rather closer to Homer Simpson (Thaler and Sunstein, 2008). Policies aimed at improving anything from individual health to personal finances, road safety, energy savings, and so on, are better designed while keeping Springfield’s most famous resident in mind. Boosted by these developments in behavioral economics, neuroeconomics has set out to open the “black box” of the economic brain (Camerer et al., 2005), progressively adding detail to an “emorational” organ (Oullier et al., 2010) constituted of neural systems so enmeshed it makes little sense to study decision-making without reference to emotional states (Sanfey et al., 2006), or—another fundamental revision to the standard models—without reference to the socio-physical environment.

## The future’s bright, the future’s interactive

The emphasis on system interaction within the organism has been accompanied by growing attention to organism-environment interaction. Given the importance of self-control to the explanation of criminal behavior (Tooby and Cosmides, 2007), research on self-regulation is particularly instructive, revealing self-control to be less of a fixed “trait” than a complex situational mechanism. How much of this resource individuals may draw on in any given circumstance is influenced by situational features, as well as individual factors. Self-control can be depleted by the prior exercise of self-control (Baumeister et al., 2007) and by the exercise of choice between alternatives (Vohs et al., 2008), with implications for the subsequent ability to self-monitor, cope with stress, control aggression, think logically, and so on. It can be depleted vicariously by watching others exercise restraint (Ackerman et al., 2009), but can also be restored vicariously by taking on the perspective of others engaged in self-control replenishing activities (Egan et al., 2012). Relevantly, self-regulatory depletion is associated with unethical behavior in well-intentioned individuals, though much less so in individuals with highly internalized moral standards, plausibly because they do not need to engage in higher cognitive processes, but automatically disregard the opportunity to behave unethically (Gino et al., 2011). This observation would seem to support situational action models of moral rule-breaking (Svensson et al., 2010).

More generally, self-regulation is sensitive to cognitive load. Decisions-making in environments which impose a high cognitive burden on individuals can lead to greater reliance on (more economical) automated decision-making, which in turn can lead to cognitive shortcuts, such as racial stereotyping (Burgess, 2010). Research into the causes of self-defeating decision-making among the poor suggests that the very conditions that define poverty, such as scarcity, impact decision-making through biosocial mechanisms which produce attentional shifts, self-control depletion, and reduce cognitive capacity generally (Spears, 2010; Shah et al., 2012; Mani et al., 2013). Self-regulation depletion also appears affected by self-belief, whereby individuals’ implicit theories of willpower moderate self-control depletion (Job et al., 2010). Overall, modern research offers an increasingly sophisticated picture of self-control as a fluctuating resource subject to the interaction of an array of individual and socio-contextual factors (see Inzlicht and Schmeichel, 2012). It also suggests avenues to integrate mechanistically so-called “root causes” (e.g., poverty) and situational choice perspectives, traditionally at odds in the context of crime studies.

Interaction is, naturally, a chief concern of those disciplines working within an adaptationist framework. In the context of evolutionary psychology, “rationality” is not portrayed as a universal construct; rather, processes are understood as domain-specific and may produce “faulty” choices when considered from another behavioral domain’s point-of-view. In this sense, rationality is not so much bounded as *ecological* (Tooby and Cosmides, 2007). This perspective suggests a framework for the continued development of still-rare ecological studies of criminal decision-making (Snook et al., 2011). It might be worthwhile in that context to explore how domain-specific processes relate (or not) to domain-general processes (Chiappe and MacDonald, 2005), as well as to niche construction (Laland and Brown, 2006).

Beyond functional explanations, evolutionary perspectives of human development have yielded constructs such as “differential susceptibility to the environment” and “biological sensitivity to context”, which add to an understanding of the role of individual differences in the outcome of person-environment interactions (Ellis et al., 2011). They suggest that heightened vulnerability to context runs both ways—some individuals are more susceptible to *both* negative *and* positive influences—and raise intriguing questions as to the persistent effect, if any, of this susceptibility into adulthood. Even these exceedingly brief examples suggest significant potential to progress CS’s take on person-situation interaction beyond its (relatively) primitive state.

## So what?

The preceding should not be taken as an entreaty for crime scientists to give up their preferred methods and reach for the fMRI—though, as with previous successful imports from epidemiology (e.g., Bowers and Johnson, 2004), greater integration will likely result in substantial methodological gains. Nor is it a demand to adopt any given approach wholesale. Indeed, the most onerous part of the conceptual shift advocated here will be to keep up with fundamental debates internal to other disciplines (e.g., Bolhuis et al., 2011). It should, however, be taken as a plea for scientific realism, for the development of theories of human behavior which go beyond axiomatic, “as-if” theoretical frameworks to specify the constellation of biosocial mechanisms which account for the phenomenon (Bunge et al., 2006). As it stands, CS’s standard model, RCP, isolates it from a wealth of knowledge in contemporary disciplines. This is a major obstacle to the development of a modern science of crime prevention.

This proposal for a more modern approach to conceptual development should not be interpreted, either, as a request to relinquish the problem-solving side of the business. Tackling practical problems generates hypotheses and throws up invaluable challenges to theoretical assumptions. Furthermore, embracing the CBN knowledge-base is bound to open up short-term avenues for crime prevention engineering. Research on the deleterious effects of cognitive load on healthcare decision-making already suggests that environmental changes, learned routines and “reflective practice” could improve the performance of crime controllers working in stressful settings (Burgess, 2010). Understanding the rewards associated with automated brain processes hints at strategies to tackle resistance to change in law enforcement organizations (Becker and Cropanzano, 2010). Experiments which elicit moral emotions such as disgust, combined with eye-tracking studies of anti-smoking warnings, could inform the design and evaluation of crime prevention publicity campaigns (see Oullier and Sauneron, 2010). Likewise, neuroimaging studies of the Ultimatum Game—which investigate why participants “irrationally” turn down money when faced with offers perceived as unfair—might help crime controllers understand why “rational” crime prevention advice is sometimes spiritedly rejected by potential victims (such as advice which suggests women should alter their behavior to prevent sexual assault).

More ambitiously, the convergence of cognitive neuroscience, social psychology, architecture (e.g., Sternberg and Wilson, 2006), consumer studies (e.g., Mick et al., 2004), and crime prevention might inspire interdisciplinary research into the design of “neurocognitively sustainable” environments, which would aim to minimize deleterious interaction (in terms of cognitive overload, depletion of self-control, and so on), with the prospect of benefit diffusion across multiple categories of social problems. The perspective of a wide-ranging contribution from evolutionary psychology has already captured the imagination of crime scientists (Roach and Pease, 2013), though reminders that adaption is an onerous explanatory concept, and that accounts of ultimate (evolutionary) causes must be accompanied by an understanding of proximal (e.g., neuropsychological) mechanisms, should be heeded (de Waal, 2002). In criminology, embryonic comparative research into the executive functioning of white collar criminals (Raine et al., 2012) hints at the possibility of tailoring prevention technologies by offending type. Executive functioning—self-regulation, but also the functions which underpin cognitive adaptability and flexibility—is likely to be a fruitful area of research for CS should it seek to account more deeply for the failure of many criminals to displace. When explaining human behavior, evaluating causal factors in isolation makes poor sense. A science of crime prevention should become comfortable with multilevel theorizing.

This paper proceeded from a simple premise: that a scientific discipline which aims to capture the imagination of future generations of researchers cannot exist only to solve practical problems; it must also set out to answer fundamental questions. While technology must be simple enough for end-users to implement, the science which is the bedrock of these technologies should be as complex as it needs to be. “Good enough” theory surrenders too much of experience to be worth the short-term benefits to any scientific discipline.

## Conflict of interest statement

The authors declare that the research was conducted in the absence of any commercial or financial relationships that could be construed as a potential conflict of interest.
